# Isolation and Characterization of Two Cellulose Morphology Mutants of *Gluconacetobacter hansenii* ATCC23769 Producing Cellulose with Lower Crystallinity

**DOI:** 10.1371/journal.pone.0119504

**Published:** 2015-03-19

**Authors:** Ying Deng, Nivedita Nagachar, Lin Fang, Xin Luan, Jeffrey M. Catchmark, Ming Tien, Teh-hui Kao

**Affiliations:** 1 Department of Biochemistry and Molecular Biology, the Pennsylvania State University, University Park, Pennsylvania, United States of America; 2 Department of Agricultural and Biological Engineering, the Pennsylvania State University, University Park, Pennsylvania, United States of America; Chang-Gung University, TAIWAN

## Abstract

*Gluconacetobacter hansenii*, a Gram-negative bacterium, produces and secrets highly crystalline cellulose into growth medium, and has long been used as a model system for studying cellulose synthesis in higher plants. Cellulose synthesis involves the formation of β-1,4 glucan chains via the polymerization of glucose units by a multi-enzyme cellulose synthase complex (CSC). These glucan chains assemble into ordered structures including crystalline microfibrils. AcsA is the catalytic subunit of the cellulose synthase enzymes in the CSC, and AcsC is required for the secretion of cellulose. However, little is known about other proteins required for the assembly of crystalline cellulose. To address this question, we visually examined cellulose pellicles formed in growth media of 763 individual colonies of *G*. *hansenii* generated via Tn5 transposon insertion mutagenesis, and identified 85 that produced cellulose with altered morphologies. X-ray diffraction analysis of these 85 mutants identified two that produced cellulose with significantly lower crystallinity than wild type. The gene disrupted in one of these two mutants encoded a lysine decarboxylase and that in the other encoded an alanine racemase. Solid-state NMR analysis revealed that cellulose produced by these two mutants contained increased amounts of non-crystalline cellulose and monosaccharides associated with non-cellulosic polysaccharides as compared to the wild type. Monosaccharide analysis detected higher percentages of galactose and mannose in cellulose produced by both mutants. Field emission scanning electron microscopy showed that cellulose produced by the mutants was unevenly distributed, with some regions appearing to contain deposition of non-cellulosic polysaccharides; however, the width of the ribbon was comparable to that of normal cellulose. As both lysine decarboxylase and alanine racemase are required for the integrity of peptidoglycan, we propose a model for the role of peptidoglycan in the assembly of crystalline cellulose.

## Introduction

Cellulose is produced not only by plants but also by microorganisms, such as algae, bacteria and fungi [1]. Various genera of bacteria, including *Gluconacetobacter* (formerly named *Acetobacter*), *Aerobacter*, *Agrobacterium*, *Rhizobium*, *Sarcina* and *Salmonella* produce so-called bacterial cellulose (BC) [2]. BC, like plant cellulose, consists of ordered glucan chains, but exhibits differing higher order molecular and nanoscale assembly resulting in unique structural and mechanical properties. For example, BC is of high purity, as it does not contain hemicelluloses, lignin or pectin, and exhibits a higher degree of crystallinity (60–90%) compared with cellulose in the plant cell wall, e.g., ~49% crystallinity for *Arabidopsis thaliana* [3] and ~32% crystallinity for cotton [4]. Depending upon the strain, however, cellulose-producing bacteria may produce hemicellulose-like extracellular polysaccharides [5], which impact cellulose organization [6].

*Gluconacetobacter* is a Gram-negative bacterium, which is capable of synthesizing large amounts of highly ordered, or crystalline, cellulose organized as twisting ribbons of microfibrillar bundles [7]. As such, *Gluconacetobacter* has long been used as a model system for studying cellulose synthesis and composite assembly in higher plants. Synthesis involves the formation of β-1,4 glucan chains which further assemble into ordered structures. From the cellulose synthase reaction, there are four distinct phases defined by sub-elementary fibrils, elementary fibrils, microfibrils, and ribbons. Images of electron micrographs showed that the surface of the *Gluconacetobacter* cell has 50–80 pore-like sites located in a regular row along the long axis of the cell, each of which is thought to secrete a 1.5 nm sub-elementary fibril composed of 10–15 glucan chains [8, 9]. It is proposed that the sub-elementary fibrils produced from more than one extrusion site aggregate to form a 3.5 nm elementary fibril [10]. The extrusions sites are sometimes grouped together, and such proximity and organization may facilitate the co-crystallization of adjacent elementary fibrils to form a 6–7 nm microfibril, which further forms bundles. Fasciation of bundles then forms the twisting cellulose ribbon (40–60 nm), which aggregates to form a cellulose pellicle produced at the top of the culture medium.

In addition to cellulose, some non-cellulosic polysaccharides, named exopolysaccharides (EPS), have been isolated from culture medium of cellulose-producing bacteria. Bacterial EPS can be classified into two groups: homo-exopolysaccharides and hetero-exopolysaccharides [11]. Homo-exopolysaccharides, similar to cellulose, dextran and levan, are made up of a single type of monosaccharide. Hetero-exopolysaccharides, similar to xanthans or gellans, are made up of several types of monosaccharides, have complex structures, and are usually synthesized inside the cell in the form of repeating units. *Gluconacetobacter* has been shown to produce a variety of water-soluble hetero-exopolysaccharides, named acetan [12,13]. Structural studies of acetan indicate that it consists of D-glucose, D-mannose, L-rhamnose, and D-glucuronic acid in a ratio of 3–4:1:1:1 [12,14]. Bacterial EPS are either attached to the cell surface by covalent bonds to form capsular polysaccharides (CPS), or are loosely associated with the cell surface [15,16]. EPS are also associated with BC; the majority of EPS can be recovered by solvent precipitation of the culture media, but a small portion of EPS cannot be separated from BC pellicles by the routine extraction protocol, and they are defined as hard to extract EPS (HE-EPS) [6]. Addition of HE-EPS (1g/L) to the culture medium disrupted the alignment of physically aggregated cellulose crystals and induced morphological changes converting a ribbon to loose bundles of cellulose microfibrils [6].

In this study, we set out to identify *G*. *hansenii* mutants that produce cellulose with reduced crystallinity, and then characterize the cellulose pellicles produced by the mutants in order to better understand the factors involved in the cellulose microfibril assembly. We report the isolation and characterization of two cellulose morphology mutants, generated by insertion of Tn5 transposon DNA in the coding region of the gene for lysine decarboxylase (LDC) and in the coding region of the gene for alanine racemase (AlaR). Based on X-ray diffraction (XRD), both mutants produced cellulose with significantly lower crystallinity than wild type. Additional analyses by solid-state NMR and monosaccharide analysis showed that the cellulose produced by these mutants contained larger amounts of non-cellulosic polysaccharides than the cellulose produced by wild type. Finally, analysis by field emission scanning electron microscopy (FESEM) showed that the cellulose produced by these mutants, unlike the cellulose produced by wild type, was unevenly distributed. We discuss how crystallization of cellulose is affected by the absence of functional LDC or AlaR.

## Materials and Methods

### Bacterial strains, plasmid, and growth media

The bacterial strains and plasmids used in this study are listed in S1 Table. A stable cellulose-overproducing clone of *G*. *hansenii* ATCC23769, previously used for Tn5 transposon mutagenesis [17], was used as the wild type control. Both wild type and mutants were grown under static and shaking conditions at 30°C in Schramm-Hestrin (SH) medium [18] for 2–3 days. Stellar competent cells of *Escherichia coli* (Clontech, Mountain View, CA) were used for transformation. Kanamycin (50 μg/ml) and tetracycline (20 μg/ml) were used to select and grow *E*. *coli* transformants, and tetracycline (20 μg/ml) and spectinomycin (100 μg/ml) were used to select and grow *G*. *hansenii* transformants. The Tn5 transposon construct was previously generated from plasmid EZ-Tn5 pMOD-3 <R6Kγori/MCS> (Epicentre Biotechnologies, Madison, WI) [17], and it contained the tetracycline resistant gene (*tetC*) from pUCD2 [19]. pGEM-T Easy vector (Promega, Madison, WI) was used for cloning and sequencing. pUCD2-Tac was generated from pUCD2, a shuttle vector between *E*. *coli* and *G*. *hansenii* [17], by ligating at the *SalI* and *PvuI* sites a 440-bp DNA fragment containing a Tac promoter, a ribosome binding site, and a transcription terminator (S1 Fig.).

### Cloning and sequencing of genes disrupted by Tn5 transposon

Genomic DNA was isolated from each morphology mutant using the Wizard Genomic DNA purification Kit (Promega), and the site of the transposon insertion was identified by the DNA Walking SpeedUp Premix Kit (Seegene Inc, Seoul, Korea) using genomic DNA as template and TS1 and TS2 provided in the kit as primers for Polymerase Chain Reactions (PCRs). All the PCR primers used in this work are listed in S2 Table. The cycling conditions were as described in Deng et al. (2013) [17]. The resulting PCR fragments were purified using the NuceloSpin Gel and PCR Clean-up Kit (Macherey-Nagel, Bethlehem, PA), and sequenced using an ABI 3730XL DNA Analyzer (Life Technologies, Carlsbad, CA) at Penn State’s Genomics Core Facility. Sequences were analyzed with the Lasergene (DNAstar, Inc. Madison, WI) software package. The nucleotide sequence of each resulting DNA fragment was compared with the genome sequence of *G*. *hansenii* ATCC23769 (GenBank accession number NZ_CM000920.1) [20] using BLASTN and BLASTtx (blast.ncbi.nlm.nil.gov).

### Complementation of cellulose morphology mutants

To make constructs to complement mutations in mutants I-23 and #52, PCRs were performed using genomic DNA of wild-type *G*. *hansenii* as template and I23-CEF/I23-CER and #52-CEF/#52-CER, respectively, as gene-specific primer (S2 Table). The resulting DNA fragments were ligated to the *Bgl*II/*Swa*I sites of pUCD2-Tac (S1 Fig.) using the In-Fusion HD Cloning kit (Clontech). The construct for complementing mutant I-23 contained the entire coding sequence, 582 bp, of the gene for lysine decarboxylase fused at the last codon with the sequence for an 8-amino acid FLAG-tag. The construct for complementing mutant #52 contained the entire coding sequence, 1,065 bp, of the gene for alanine racemase fused at the last codon with the same sequence for the FLAG-tag. Both constructs were transformed into *E*. *coli* Stellar competent cells, and the transformants were selected on LB agar plates containing kanamycin. The plasmid DNA purified from a transformant of each construct was electroporated into the mutant in which the respective gene was disrupted by the Tn5 transposon insertion. The transformed cells were grown in SH medium with cellulase (0.02%) overnight at 30°C, and the cells were resuspended and plated on SH agar plates containing spectinomycin.

### Immunoblotting analysis

For immunoblots with anti-FLAG antibody and with anti-AcsB,-AcsC, and -AcsD antibodies [21], isolation of total protein, conditions for SDS-polyacrylamide gel electrophoresis, and transfer of proteins onto Immobilon PVDF membranes (EMD Millipore, Darmstadt, Germany) were as described by Deng et al. (2013) [17]. For immunoblot with anti-AcsA_cat_ antibody [21], the only difference was that total protein was incubated in lithium dodecyl sulfate sample buffer on ice for one hour, instead of being boiled in the standard SDS sample buffer. The membranes were blotted using anti-FLAG antibody (1:1000; Sigma-Aldrich, St. Louis, MO) and anti-AcsA_cat_,-AcsB,-AcsC, and -AcsD antibodies (1:500 dilution) as the primary antibodies. The secondary antibodies used were anti-mouse IgG conjugated with alkaline phosphatase (1:15,000 dilution; Sigma-Aldrich) for the blot with anti-FLAG antibody, and anti-rabbit IgG conjugated with alkaline phosphatase (1:15,000 dilution; Sigma-Aldrich) for all the other blots.

### Preparation of cellulose pellicles

The mutants and wild type were grown in 100 ml SH medium under static conditions for 7 days. Cellulose pellicles, formed at the surface of each medium, were harvested, washed with 0.1 M NaOH at 80°C twice, one hour each, with gentle stirring, and then washed with distilled water until the pH dropped to 7. The pellicles were freeze-dried, and either directly used for measurement or stored at room temperature until use. After each mutant had been complemented with a functional copy of the gene disrupted by Tn5 transposon DNA, the complemented mutant was grown in 100 ml SH medium under static conditions for 7 days. Cellulose pellicles were similarly processed.

### X-ray diffraction (XRD)

XRD diffractograms were recorded using PANalyticalX’Pert Pro multi-purpose diffractometer with Cu Kα radiation generated at 45 kV and 40 mA. The diffractometer was used in reflection mode with the automatic divergence slit. The data were collected in the 2θ range 5–40° with a step size of 0.026°. Freeze-dried BC samples were mounted onto a quartz sample holder. A pseudo Voigt function was used to profile the peak shape and area, assuming a linear background. A broad peak at around 21.5° was assigned to the amorphous contribution. Crystallinity is calculated from the ratio of the area of all crystalline peaks to the total area [22].

### Solid-state NMR

^13^C ramp-CPMAS spectra with total sideband suppression (TOSS) were measured on a Bruker AV300 WB spectrometer at 75.55 MHz with a 4-mm H/X CPMAS probe. Approximately 80 mg of sample was placed in a zirconia rotor and the MAS set to 5 kHz. Acquisition conditions: spectral width of 38 kHz, acquisition time of 13.6 ms with Spinal-128 ^1^H decoupling during acquisition, recycle delay of 3 s, contact time of 2 ms, proton 90° pulse of 5.2 μs (@ -1.2 dB), and number of scans being 56k. Processing: FID was zero-filled to 8k points, and multiplied by exponential function with LB = 50Hz.

### Monosaccharide analysis

The monosaccharide composition of cellulose pellicles prepared as described above was determined after hydrolysis in 72% H_2_SO_4_ at 30°C for 1 h, followed by diluting the H_2_SO_4_ solution to 4% and autoclaving for 1 h, then neutralized with 0.1 N NaOH [23]. Analysis of monosaccharides of the hydrolysates was performed on a Dionex ICS-5000 Capillary Reagent-Free IC System (Dionex, Sunnyvale, CA) and a CarboPac 20 column (Dionex). The following standards were run under the same conditions to determine the retention times and response factors: arabinose, rhamnose, fructose, galactose, glucose, xylose, mannose, glucuronic acid and galacturonic acid. Monosaccharides were identified by comparing their retention times to the monosaccharide standards.

### Field emission scanning electron microscopy (FESEM)

The FESEM images were collected on uncoated lyophilized BC samples using a Zeiss Merlin Field Emission Scanning Electron Microscope (Carl Zeiss NTS GmHB, Oberkochen, Germany) operated at 3 kV, located at the Materials Research Institute of the Penn State University.

### Light microscopy

Cells of wild type and mutants I-23 and #52 were grown in 5 ml SH medium containing cellulose (0.02%) for 2 days under shaking conditions. For each sample, a drop of 10-μl culture was placed on a polylysine coated slide and allowed to air dry; a cover glass was then placed on the slide. The slides were observed using a 100x oil objective lens of an Olympus BX63 microscope system (Olympus, Tokyo, Japan).

## Results

### Isolation of cellulose morphology mutants generated

To identify *G*. *hansenii* genes that are involved in the synthesis, secretion, assembly, and crystallization of cellulose, we previously used Tn5 transposon random mutagenesis to generate mutants that might be defective in any of the steps. This was accomplished by transforming competent cells of wild-type *G*. *hansenii* with a complex of Tn5 transposon DNA (containing the *tetC* gene from pUCD2) and EZ-Tn5 transposase, followed by selecting transformants on SH agar plates containing tetracycline [17]. A total of 769 individual colonies were grown in shaking cultures in 5 ml SH medium with tetracycline for 2 days. We previously reported the identification and characterization of six mutants that failed to produce or secrete cellulose into the medium [17]. Among the 763 colonies examined in this study, 85 produced cellulose that appeared abnormal after 2 days of growth under shaking conditions. Cellulose produced by wild type appeared as a round pellicle at the bottom of the culture under shaking conditions and the medium remained clear. Cellulose produced by 19 of these 85 mutants and wild type is shown in S2 Fig. Also shown are two of the previously characterized non-cellulose-producing mutants [17] serving as negative controls.

**Fig 1:**
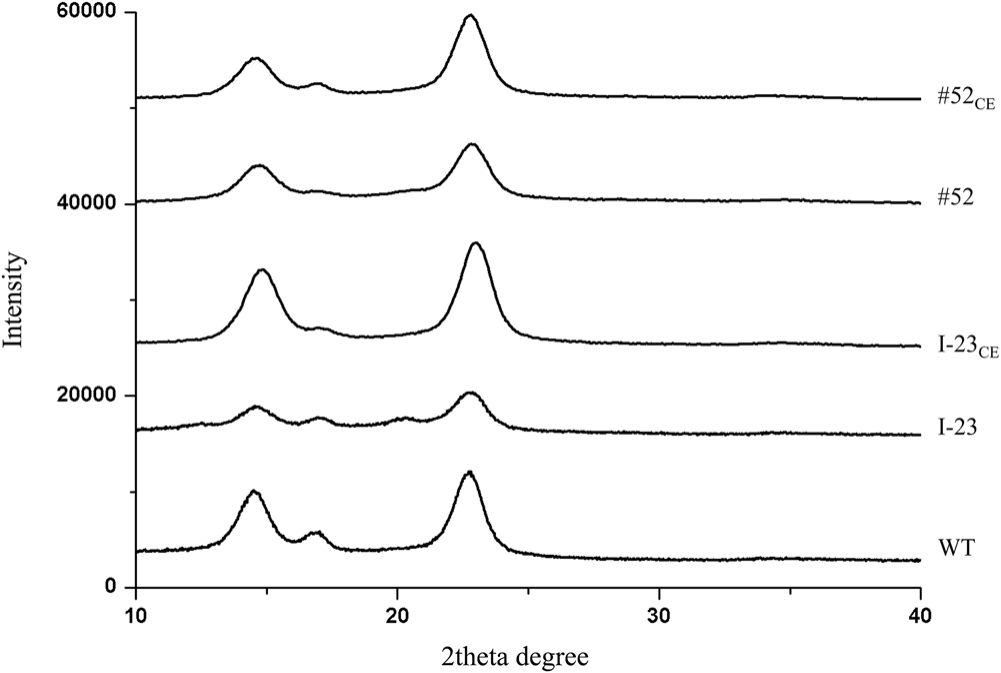
XRD diffractograms of cellulose morphology mutants I-23 and #52, their complemented transformants, I-23_CE_ and #52_CE_, and wild type. For each mutant, complemented transformant and wild type, cellulose pellicles prepared from three biological replicates were used for analysis, and the result of one representative diffractogram is shown.

Each of these 85 mutants was grown in a 100-ml medium with tetracycline for 7 days under static conditions, and the cellulose pellicles produced were analyzed by XRD. For the colonies that were found to produce cellulose with lower crystallinity than wild type, we further examined two additional biological replicates of each colony, as well as wild type, to ensure reproducibility. We identified two mutants, named I-23 and #52, which reproducibly produced cellulose with significantly lower crystallinity than wild type. The XRD diffractograms of one of the three biological replicates of these two mutants and wild type are shown in Fig. 1. The mean of the degree of crystallinity for the three biological replicates of mutant I-23 was 70.5±0.9%; the mean of the degree of crystallinity for the three biological replicates of mutant #52 was 59.7±2.3%. In contrast, the mean of the degree of crystallinity for the three biological replicates of wild type was 81.4±1.2%.

### Identification of genes disrupted in two cellulose morphology mutants

We used a PCR-based genomic DNA walking method, as described in the Materials and Methods section, to identify the Tn5 transposon insertion sites in mutants I-23 and #52, and the results are summarized in Table 1. Mutant I-23 had Tn5 transposon DNA inserted in the coding region of the gene for lysine decarboxylase (LDC) (accession number EFG85846; 194 amino acids), resulting in truncation of the C-terminal 79 amino acids. Mutant #52 had Tn5 transposon DNA inserted in the coding region of the gene for alanine racemase (AlaR) (accession number EFG85940; 355 amino acids), resulting in truncation of the C-terminal 146 amino acids.

**Table 1:** Genes disrupted by Tn5 transposon DNA in cellulose morphology mutants.

Mutant	Gene disrupted	Protein encoded	^a^ Insertion site	Full coding sequence
I-23	*LDC*	Lysine decarboxylase	347–348 bp	582 bp
#52	*AlaR*	Alanine racemase	628–629 bp	1065 bp

^a^ Counting from the translation initiation codon.

The genes flanking *LDC* encode an MFS (Major Facilitator Superfamily) transporter (49 bp upstream; transcribed in the same direction as *LDC*) and a ferrochelatase (1,874 bp downstream; transcribed in the opposite direction as *LDC*). Considering the distance between *LDC* and the gene encoding ferrochelatase, Tn5 insertion in *LDC* most likely did not affect its expression. It is not known whether *LDC* and *MFS* are located in the same operon in *G*. *hansenii*, but considering that *MFS* is upstream of *LDC*, Tn5 insertion in *LDC* most likely did not affect its expression. For example, in our previous study of the *Acs* operon (consisting of *AcsA*, *AcsB* and *AcsC*) of *G*. *hansenii*, we found that Tn5 insertion in *AcsC* did not affect the production of its upstream AcsA or AcsB [17]. The genes flanking *AlaR* encode a nitrilase/cyanide hydratase (555 bp upstream; transcribed in the same direction as *AlaR*) and a putative cytoplasmic protein (979 bp downstream; transcribed in the same direction as *AlaR*). Judging from the distance between *AlaR* and these two flanking genes, Tn5 insertion in *AlaR* most likely did not affect the expression of either.

### Complementation of the morphology mutants

To verify that the cellulose morphology phenotype of mutants I-23 and #52 was indeed caused by insertion of Tn5 transposon DNA in the *LDC* and *AlaR* genes, respectively, we used a *Tac* promoter to express the coding sequence of a functional copy of *LDC* in mutant I-23 and the coding sequence of a functional copy of *AlaR* in mutant #52 to determine whether the phenotype could be complemented. Each construct in pUCD2-Tac was introduced into the respective mutant via electroporation, and the transformants were selected on SH agar plates containing spectinomycin. To determine the crystallinity of cellulose produced by the complemented mutants I-23_CE_ and #52*CE*, we inoculated each of three independent colonies in 100-ml SH medium containing both tetracycline and spectinomycin, grew the cells under static conditions for 7 days, and processed the cellulose pellicles for XRD analysis. The results are shown in Fig. 1. For I-23_CE_, the mean of the crystallinity of cellulose produced by the three independent colonies was 77.4±0.8%, higher than the 70.5±0.9% of mutant I-23 and close to the 81.4±1.2% of wild type. For #52_CE_, the mean of the crystallinity of cellulose produced by the three independent colonies was 82.6±1.7%, higher than the 59.7±2.3% of mutant #52, and comparable to the 81.4±1.2% of wild type.

**Fig 2:**
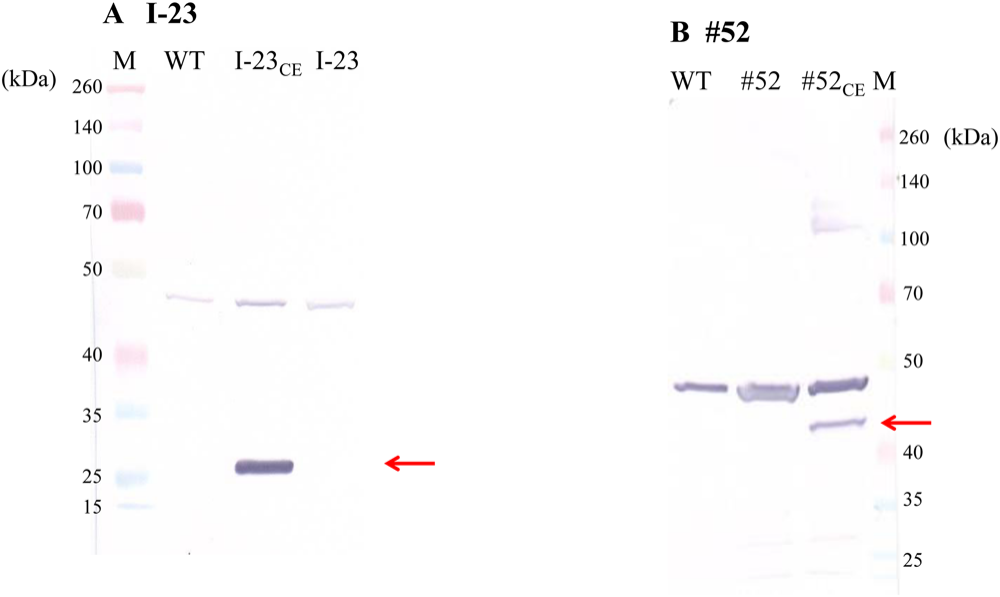
Immunoblotting analysis of production of LDC:FLAG-tag (A) and AlaR:FLAG-tag (B). Total protein (40 μg) was loaded in each lane of 12% SDS-polyacrylamide gels, and anti-FLAG was used to detect the proteins produced. Arrows indicate LDC:FLAG-tag (A) and AlaR:FLAG-tag (B). M: markers for molecular masses. WT: wild type control

We then used anti-FLAG antibody to examine the level of LDC produced by one of the three colonies of I-23_CE_ and the level of AlaR produced by one of the three colonies of #52_CE_ by immunoblotting. A protein band close to the expected molecular mass of LDC-FLAG (~23 kDa) was detected in I-23_CE_, but not in wild type or I-23 (Fig. 2A). A protein band of the expected molecular mass of AlaR-FLAG (~44 kDa) was detected in #52_CE_, but not in wild type or #52 (Fig. 2B). Thus, the results from the complementation experiment confirmed that the cellulose morphology phenotype of mutants I-23 and #52 was caused by the insertion of Tn5 transposon DNA in *LDC* and *AlaR*, respectively.

### Cellulose production by the morphology mutants and complemented mutants

Before comparing the amounts of cellulose produced by wild type, mutants I-23 and #52, and complemented mutants I-23_CE_ and #52_CE_, we examined whether disruption of *LDC* and *AlaR* might affect the growth rate of I-23 and #52, respectively. Cells of wild type, I-23, #52, I-23_CE_ and #52_CE_ were grown in 100 ml SH medium containing cellulase (0.02%) under both static and shaking conditions; for I-23 and #52, tetracycline (20 μg/ml) was added; and for I-23_CE_ and #52_CE_, both tetracycline (20 μg/ml) and spectinomycin (100 μg/ml) were added. The growth of each culture was monitored daily by its absorbance at 600 nm for 10 days under shaking conditions and 15 days under static conditions. The growth rates of all these cells were quite similar under both growth conditions (S3 Fig.). We then grew cells of wild type, the two mutants, and their complemented mutants in 5 ml SH medium containing cellulase (0.02%) for two days, and then used equal numbers of cells from each culture, based on absorbance at 600 nm, to inoculate 100 ml SH medium. After growth under static conditions for 7 days, cellulose pellicles were harvested, and their dry weights were determined. For mutant I-23, the mean of the amount of cellulose produced by three independent colonies was 34.9±1.5% that of wild type. For mutant #52, the mean of the amount of cellulose produced by three independent colonies was 25.0±4.9% that of wild type. As the growth rates of mutants I-23 and #52 were similar to that of wild type, these results suggest that disruption of LDC and AlaR affected not only the morphology, but also the amount, of cellulose produced. We then examined whether the reduction in cellulose production in these two mutants was caused by lower than wild type levels of any of the known components of the cellulose synthase complex (CSC), AcsA, AcsB, AcsC and AcsD [24]. The results of immunoblotting showed that absence of functional LDC or AlaR did not affect the level of any of these proteins (S4 Fig.).

For I-23_CE_, the mean of the amount of cellulose produced by three independent colonies was 77.1±0.9% that of wild type. For #52_CE_, the mean of the amount of cellulose produced by three independent colonies was 94.1±2.4% that of wild type. Thus, the cellulose producing ability of I-23 and #52 was largely restored in their complemented mutants.

### Solid-state NMR (CP-TOSS) analysis

**Fig 3:**
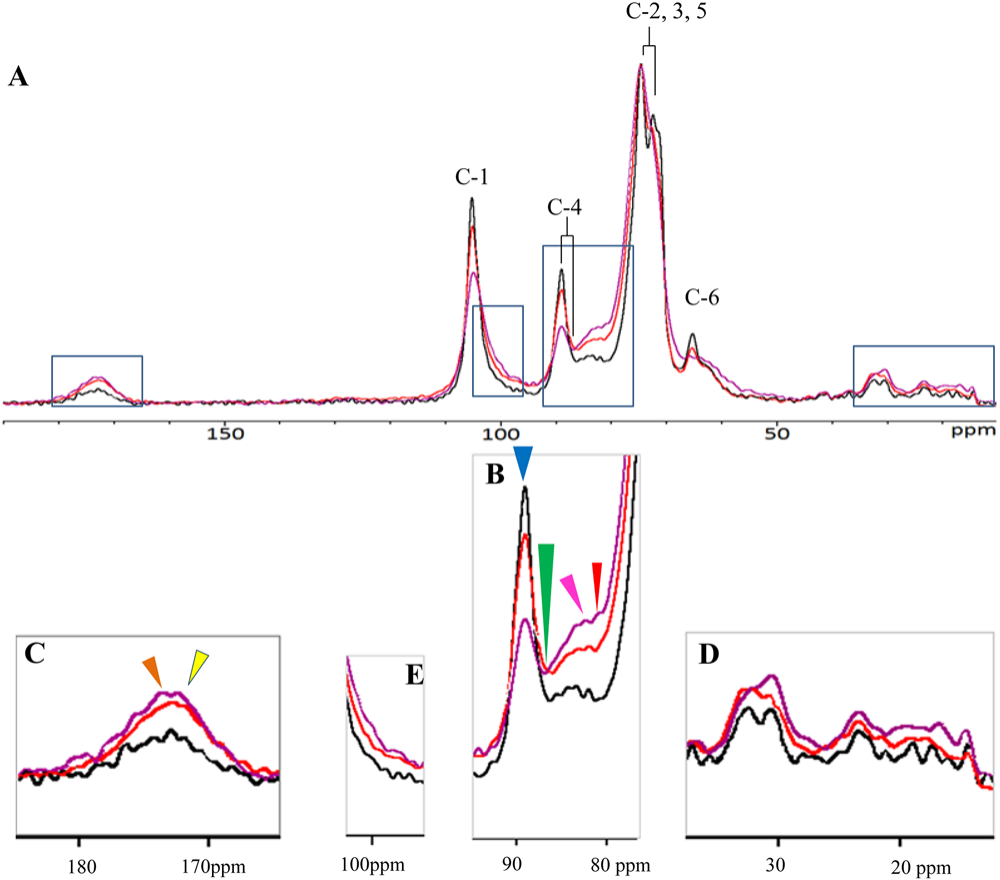
^13^C NMR spectra of cellulose pellicles produced by mutant I-23 (red), mutant #52 (purple), and wild type (black). (A) Full spectra with carbon-1 to carbon-6 of glucose in crystalline cellulose indicated. (B) The region of the spectra showing the peak area of carbon-4 of glucose in crystalline cellulose (88.9 ppm), indicated by a blue arrow; peak area of carbon-4 of glucose in non-crystalline cellulose (85.0 ppm), indicated by a green arrow; peak areas of 82.0 ppm, indicated by a red arrow, and 83.0 ppm, indicated by a pink arrow, assigned to carbons of monosaccharides in non-cellulosic polysaccharides. (C) The region of the spectra showing the peak areas of 172.8 ppm, indicated by a yellow arrow, and 176.3 ppm peak, indicated by an orange arrow, assigned to ester or acid carboxyl carbons of monosaccharides in non-cellulosic polysaccharides. (D) The region of the spectra showing peaks assigned to methyl groups at ~20 ppm. (E) The region of the spectra showing carbon-1 of monosaccharides in non-cellulosic polysaccharides.

CP-TOSS was used to further examine the differences between the cellulose produced by wild type and mutants I-23 and #52. The full spectra are shown in Fig. 3A. The peaks for all six carbons of the glucose unit in crystalline cellulose are indicated by the carbon numbers C-1 to C-6. These peaks were the major peaks observed with the wild type and both mutant samples. Carbon-4 of crystalline cellulose has a peak at 88.9 ppm, whereas this carbon of non-crystalline cellulose has a peak at 85.0 ppm. The ratio of these two peak areas is typically used to assess degree of cellulose crystallinity [25]. This ratio (blue arrow for 88.9 ppm and green arrow for 85.0 ppm; Fig. 3B) is higher for wild type than for both mutants. However, as described below, we could not use the ratio of these two peak areas to accurately determine the crystallinity of each cellulose sample, as the peak of carbon-4 of glucose in non-crystalline cellulose was quite close to the 82.0 ppm and 83.0 ppm peaks.

The peaks at 82.0 ppm and 83.0 ppm, indicated by red and pink arrows, respectively (Fig. 3B), may be assigned to carbons of hexoses of non-cellulosic polysaccharides. However, they are located in a region with many other peaks, as peaks of carbon-2 and carbon-4 of many hydrocarbon compounds are in this area, e.g., carbon-2 (82.0 ppm) and carbon-4 (84.9 ppm) of arabinose in pectin [26], and carbon-4 of glucose (83.0 ppm) in xyloglucan [27]. Although these peaks could not be specifically assigned, their presence suggests that there were non-cellulosic polysaccharides present in cellulose produced by wild type and both mutants. The peaks for both mutants were higher than those for wild type, suggesting that there were larger amounts of non-cellulose polysaccharides present in both mutants than in wild type.

We also examined the 172.8 ppm and 176.3 ppm peaks, which have been assigned to ester or acid carboxyl carbons. This region of the three spectra is enlarged in Fig. 3C, with the 172.8 ppm and 176.3 ppm peaks indicated with yellow and orange arrows, respectively. These peaks have been assigned as protein peaks in some NMR studies of hydrocarbonates [28]. However, ester or acid carboxyl carbons are also present in monosaccharides found in certain polysaccharides, e.g., carbon-6 of GalA (171.3 ppm) in pectin [29] and the carbonyl carbon of the acetyl group (174.4 ppm and 174.9 ppm) in acetan [30]. These peaks were higher in both mutants than in wild type (Fig. 3C), suggesting that there are larger amounts of non-cellulosic polysaccharides in both mutants than in wild type. Moreover, the existence of acetyl groups would suggest that there should be methyl groups at ~20 ppm. This region of the three spectra is enlarged and shown in Fig. 3D. The peak heights associated with both mutants were greater than wild type, consistent with our interpretation of the 172.8 ppm and 176.3 ppm peaks.

The existence of non-cellulosic polysaccharides would also suggest that carbon-1 of the monosaccharide subunits in non-cellulosic polysaccharides should be observed at a higher field than carbon-1 of glucose in cellulose, e.g., this carbon of rhamnose and GalA in RG-I is at 101.0 ppm [27, 31], of Gal in xyloglucan is at 104.0 ppm [27], and of xylose in xyloglucan is at 99.7 ppm [32]. This area, enlarged in Fig. 3E, was greater in both mutants than in wild type.

In summary, the results from the solid-state NMR analysis suggest that the cellulose pellicles produced by both mutants contained larger amounts of non-crystalline cellulosic glucose and larger amounts of non-cellulosic polysaccharides than the cellulose pellicle produced by wild type.

### Monosaccharide analysis

To further confirm the existence of non-cellulosic polysaccharides in cellulose produced by the two mutants, the monosaccharide content of the cellulose pellicles produced by the mutants and wild type were analyzed by ion chromatography (Dionex). Cellulose pellicles were treated as described in the Materials and Methods section, including an alkaline treatment, which served to remove most of the impurities. However, this treatment was not able to remove a fraction of the EPS (referred to as HE-EPS) [6]. The ion chromatography analysis identified glucose, galactose and mannose as the monosaccharides released from both mutant and wild-type pellicles (Table 2). Glucose was the most abundant monosaccharide released from each sample. The percent weight of galactose and mannose was at least two-fold higher in the two mutants when compared to wild type. This suggests that cellulose produced by these two mutants contained larger amounts of non-cellulosic polysaccharides.

**Table 2:** Monosaccharides released from acid-treated cellulose pellicles produced by wild type and two cellulose morphology mutants.

Monosaccharide	Wild type	I-23	#52
Galactose (%)^a^	0.3	0.8	0.6
Glucose (%)	98.1	95.6	95.4
Mannose (%)	1.5	3.6	3.9

^a^ Percent total weight

### Cellulose morphology analysis

**Fig 4:**
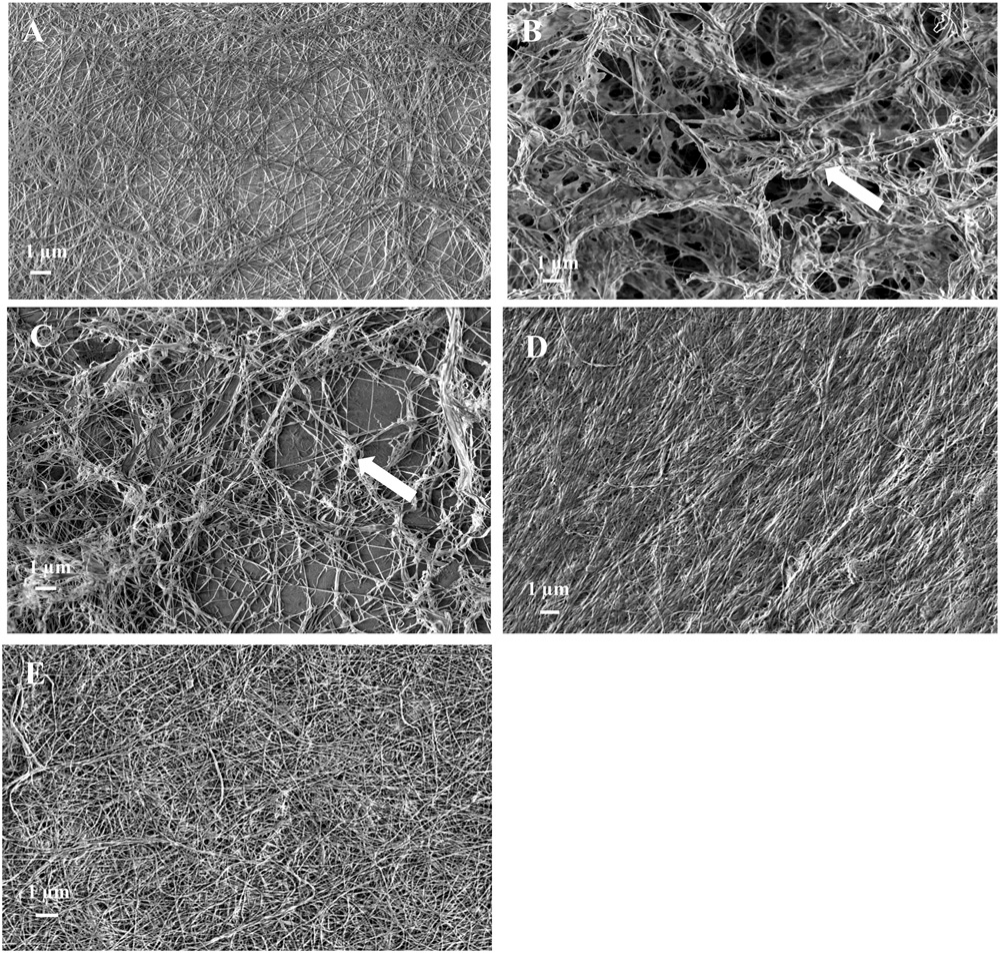
FESEM images of cellulose produced by wild type (A), mutant I-23 (B), mutant #52 mutant (C), complemented I-23 (I-23_CE_; D), and complemented #52 (#52_CE_; E). White arrows in **(B)** and **(C)** point to a region of cellulose pellicles produced by mutant I-23 and mutant #52 where non-cellulosic polysaccharides may be embedded.

Field emission scanning electron microscopy (FESEM) was performed to observe cellulose morphology and microstructure. The images of the freeze-dried purified cellulose pellicles of wild type, both mutants, and the complemented mutants are shown in Fig. 4. Cellulose produced by wild type showed a uniformly distributed, densely packed, network of randomly oriented ribbons ranging from 30 to 90 nm in diameter (Fig. 4A). This agrees with the typical morphology reported for BC [33, 34]. In contrast, the network of cellulose produced by mutants I-23 (Fig. 4B) and #52 (Fig. 4C) was unevenly distributed, with some regions appearing to contain local deposition of non-cellulosic polysaccharides. The cellulose fibrils might be buried within these regions. The average ribbon width calculated from 50 individual cellulose ribbons shown in Fig. 4B and Fig. 4C was ~50 nm, which was comparable to that of cellulose produced by wild type. For the complemented mutants I-23_CE_ (Fig. 4D) and #52_CE_, (Fig. 4E), the local deposition of non-cellulosic polysaccharides disappeared and the network of cellulose resembled that of wild type. These results further support the notion that disruption of *LDC* and *AlaR* is responsible for the cellulose morphology phenotype in mutants I-23 and #52, respectively.

## Discussion

In this work, we screened the previously generated Tn5 transposon insertion mutants of *G*. *hansenii* ATCC23769 for those that produced cellulose with altered morphologies. Based on visual inspection of cellulose pellicles formed in the growth medium, most of the 763 individual colonies examined produced cellulose with normal morphology. The 85 colonies that produced cellulose with abnormal morphologies were further analyzed by X-ray diffraction (XRD). We found two colonies that reproducibly produced cellulose with significantly lower degrees of crystallinity than wild type (59.7±2.3% for mutant #52 and 70.5±0.9% for mutant I-23, vs. 81.4±1.2% for wild type). The gene encoding lysine decarboxylase (LDC) is disrupted in mutant I-23 and the gene encoding alanine racemase (AlaR) is disrupted in mutant #52, neither of which has previously been implicated in the assembly of crystalline cellulose. That LDC and AlaR are required for assembly of crystalline cellulose was confirmed by the complementation experiments. Introducing a functional copy of each gene into its respective mutant restored the crystallinity of the cellulose produced to similar degrees as wild type. Solid-state NMR corroborated the results from XRD in that it indicated decreased crystallinity for the cellulose produced by both mutants. The NMR results also indicated that the mutants produced higher amounts of non-cellulosic monosaccharides, e.g., those containing ester or acid carboxyl carbons. Monosaccharide analysis confirmed the presence in both mutants of higher percentages of two non-cellulose monosaccharides, galactose and mannose, than in wild type.

Thus, the results from XRD, solid-state NMR and monosaccharide analyses taken together suggest that lack of a functional LDC in mutant I-23 and lack of a functional AlaR in mutant #52 result in the incorporation of larger than normal amounts of non-cellulosic polysaccharides into the cellulose produced and a reduction in cellulose crystallinity. The degree to which each of these factors participates in the reduction of crystallinity is not clear. The presence of additional non-cellulosic polysaccharide content could impact cellulose crystallinity, like in the case of BC grown in the presence of xyloglucan [34], although that had not been found in other cellulose producing strains. A recent study by Fang and Catchmark (2014) [6] showed that adding HE-EPS produced by *G*. *xylinus* strain ATCC 53582 back into the culture media disrupted crystal alignment without impacting crystallization or co-crystallization processes. Thus it appears that some non-cellulosic polysaccharides may interact with cellulose without interfering with crystallization. The presence of additional non-cellulosic polysaccharides may have resulted from metabolic factors associated with the substantial reduction in cellulose production allowing more non-cellulosic polysaccharides to be produced.

**Fig 5:**
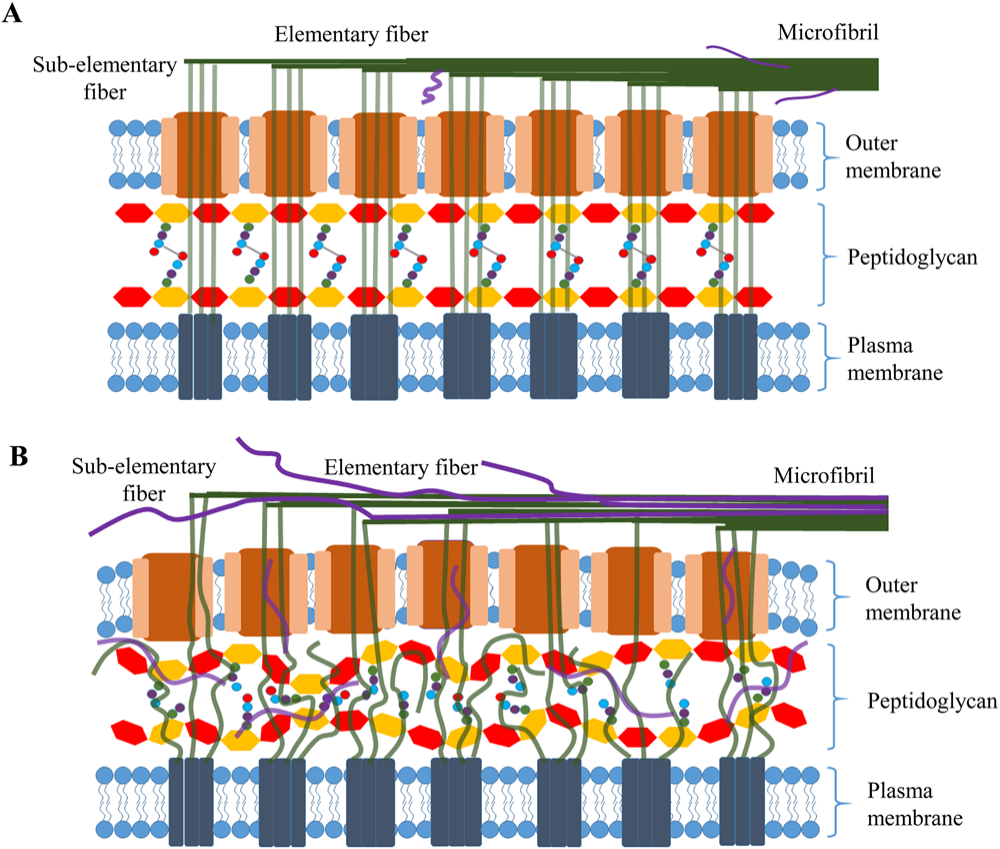
Model for the role of the peptidoglycan framework in cellulose synthesis. (A) The peptidoglycan framework providing guidance of glucan chains (green line) produced by each cellulose synthase complex located in the inner membrane through the periplasm to a pore, located in the outer membrane and in registry, to form a sub-elementary fiber for extrusion. Several sub-elementary fibers produced from more than one extrusion site aggregate to form a 3.5 nm elementary fibril, and adjacent elementary fibrils co-crystallize to form a 6–7 nm microfibril. (B) Effects of disruption of the peptidoglycan framework on assembly of glucan chains. The glucan chains (green line), synthesized in the inner membrane, may not be in perfect registry with the pore, and lack of guidance may result in some glucan chains not participating in the assembly of a sub-elemental fibril. Thus, the number of glucan chains that reach a pore may be reduced, and the less tightly packed glucan chains in the periplasm may allow non-cellulosic polysaccharides (purple curve line) to be incorporated into the sub-elementary fibril.

Both LDC and AlaR have an important role in the establishment of the peptidoglycan framework in Gram-negative bacteria (Fig. 5A). The peptidoglycan cell wall consists of linear chains of alternating beta 1,4-linked N-acetylglucosamine and N-acetylmuramic acid. The lactic acid residue of N-acetylmuramic acid forms an amide bond with a D-amino-acid-containing tetrapeptide. Neighboring chains are cross-linked through their tetrapeptide side chains in a variety of ways. One way is through direct amide linkage between one of the amino acids in each tetrapeptide. LDC converts lysine to cadaverine, a diamine, which has been shown in some bacteria to covalently link to the D-isoglutamate, the second amino acid in the tetrapeptide [35].

**Fig 6:**
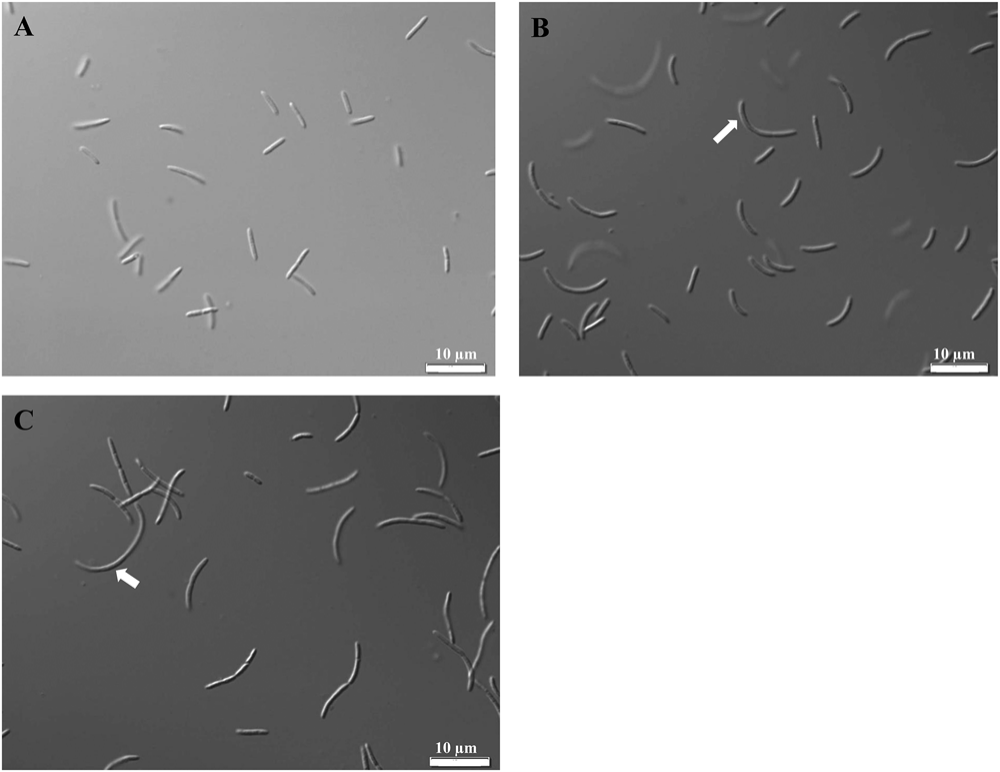
Microscopic images of cells of wild type (A), mutant I-23 (B) and mutant #52 mutant (C). Wild-type cells are rod-shaped, whereas cells of both mutants are curve-shaped, indicated by white arrows. The magnitude of magnification is 100x.

This linkage is essential for the integrity of the cell wall. Another D-amino acid in the tetrapeptide is D-alanine, which is converted from L-alanine by AlaR and is required for crosslinking of polysaccharide chains of peptidoglycan. Thus, absence of either LDC or AlaR is expected to affect the integrity of peptidoglycan. Microscopic examination of I-23, #52 and wild-type cells showed that both mutants had very different cell morphology than wild type. Unlike the rod-shaped wild-type cells (Fig. 6A), the cells of both mutants were curved (Fig. 6B and Fig. 6C). This is consistent with defects in peptidoglycan, which is required to maintain structural strength of the cell and counteract the osmotic pressure of the cytoplasm [36].

While our results indicate that LDC and AlaR are required for cellulose crystallinity, the precise mechanism is not known. Nevertheless, it is apparent that cell wall integrity impacts cellulose crystallinity. A possible mechanism is illustrated in Fig. 5A. During the first phase of cellulose synthesis, β-1–4-glucan chains are synthesized by the catalytic component of each cellulose synthase complex (CSC) located in the cytoplasmic membrane, and the glucan chains traverse the periplasmic space to the outer membrane for extrusion. It is thought that 10–15 glycan chains arrive at the same pore located in the outer membrane to form a 1.5 nm sub-elementary fibril. Several fibers, produced from more than one extrusion site, then aggregate to form a 3.5 nm elementary fibril. Crystallization of several elementary fibrils then forms a 6–7 nm cellulose microfibril. An intact peptidoglycan framework may be essential for guiding glucan chains, allowing maximum interactions between them in their journey to the outer membrane and ensuring their being in registry with a pore for extrusion. Disruption of the peptidoglycan framework affects the cell morphology (Fig. 6) and may result in the glucan chains not being in perfect registry with the pore (Fig. 5B). Moreover, not having the peptidoglycan framework to guide the glucan chains, some glucan chains might trail other glucan chains and fail to “participate” in the assembly of a sub-elementary fibril. Thus, even though absence of functional LDC or AlaR does not affect the levels of any of the known components of the CSC (S4 Fig.), the number of glucan chains that reach a pore may be reduced. This can explain our finding that the amount of cellulose produced by each morphology mutant is significantly lower than wild type (~1/3 wild-type level for mutant I-23, and ~1/4 wild-type level for mutant #52). In addition, the less tightly packed glucan chains in the periplasm may allow non-cellulosic polysaccharides to be incorporated into the sub-elementary fibril (Fig. 5B). It is also possible that non-cellulosic polysaccharides are incorporated at later stages of cellulose synthesis, prior to crystallization of cellulose, as it has been shown that incorporation of HE-EPS occurs during cellulose synthesis, but does not occur after crystallization of BC [6]. As the width of the cellulose ribbons produced by both mutants is comparable to that of wild type, disruption of the peptidoglycan framework may facilitate the incorporation of non-cellulosic polysaccharides, but does not appear to have a significant effect on the final stage of individual ribbon assembly.

It remains to be determined what is the nature of the non-cellulosic polysaccharides embedded in the cellulose produced by mutants I-23 and #52. As mannose, but not rhamnose or glucuronic acid, was detected in the monosaccharide analysis, these non-cellulosic polysaccharides may be different from the previously reported water-soluble EPS, acetan, which also contains rhamnose and glucuronic acid. Interactions of EPS with cellulose may occur through the mannan or cellulosic backbone of EPS. Strong interactions between mannose-based polysaccharides and cellulose were predicted by Whitney et al. (2006) [33] because the mannose backbone is stereochemically similar to cellulose, differing only in the configuration of the hydroxyl group at carbon-2. Chanzy (1982) [37] showed that glucomannan extracted from ivory nut adopted a two-fold screw conformation closely matching that of cellulose; this was confirmed by NMR analysis of glucomannan/cellulose composites [15]. The effect of non-cellulosic polysaccharides on cellulose crystallinity in *Gluconcetobacter* may be similar to the effect of hemicellulose and pectin on cellulose in the plant cell wall. Thus, the cellulose morphology mutants studied here may serve as a model for studying the interactions between non-cellulosic polysaccharides and cellulose, and how these interactions affect the assembly of crystalline cellulose.

Finally, this work, along with our previous work [17], has demonstrated the feasibility of using Tn5 transposon random mutagenesis, a forward genetic approach, to identify genes involved in the synthesis and assembly of crystalline cellulose by *Gluconacetobacter*. Considering that a large number of the cellulose morphology mutants we have identified are yet to be further studied, there may be more genes that are either directly or indirectly involved in the assembly of crystalline cellulose.

## Supporting Information

S1 Fig Schematic of pUCD2-Tac used for complementation experiment. The sequences of two of the regions inserted into pUCD2 are shown below the plasmid. Genes encoding lysine decarboxylase and alanine racemase are separately ligated into the *Bgl*II and *Swa*I restriction enzyme sites for expression in mutants I-23 and #52, respectively. Tacp: Tac promoter; RBS: ribosome binding site; Terminator, transcription terminator.
(TIF)

S2 Fig Cellulose produced by mutants I-23 and #52, as well as by some of the other morphology mutants generated by Tn5 transposon mutagenesis. Cellulose produced was examined after growth in medium for 2 days under shaking conditions. WT: wild type; I-7 and I-13: non-cellulose-producing mutants used as negative controls [17].
(TIF)

S3 Fig Growth curves of wild type (WT), mutants I-23 and #52, and complemented mutants I-23_CE_ and #52_CE_ under static (A) and shaking (B) conditions. Cells were grown in 100 ml SH medium with cellulase (0.02%) under static conditions for 15 days and under shaking conditions for 10 days; for I-23 and #52, tetracycline (20 μg/ml) was also added to the medium; and for I-23_CE_ and #52_CE_, both tetracycline (20 μg/ml) and spectinomycin (100 μg/ml) were added to the medium. At the same time on each day, OD_600_ values were taken for each culture.
(TIF)

S4 Fig Immunoblotting analysis of AcsA, AcsB, AcsC and AcsD of the cellulose synthase complex produced by wild type (WT) and mutants, I-23 and #52. Total protein (40 μg) was loaded in each lane of 12% SDS-polyacrylamide gels. The antibody used for each blot is shown to the left, and the expected molecular mass of each protein is shown to the right.
(TIF)

S1 Table Bacterial strains and plasmids used in this study. (PDF)

S2 Table Primers used in this study. (PDF)
