# Supplementary figures and images for: Isolation and Characterization of Two Cellulose Morphology Mutants of Gluconacetobacter hansenii ATCC23769 Producing Cellulose with Lower Crystallinity
Source: PLoS One. 2015 Mar 19;10(3):e0119504. doi: 10.1371/journal.pone.0119504 (PMC4366249; doi:10.1371/journal.pone.0119504)

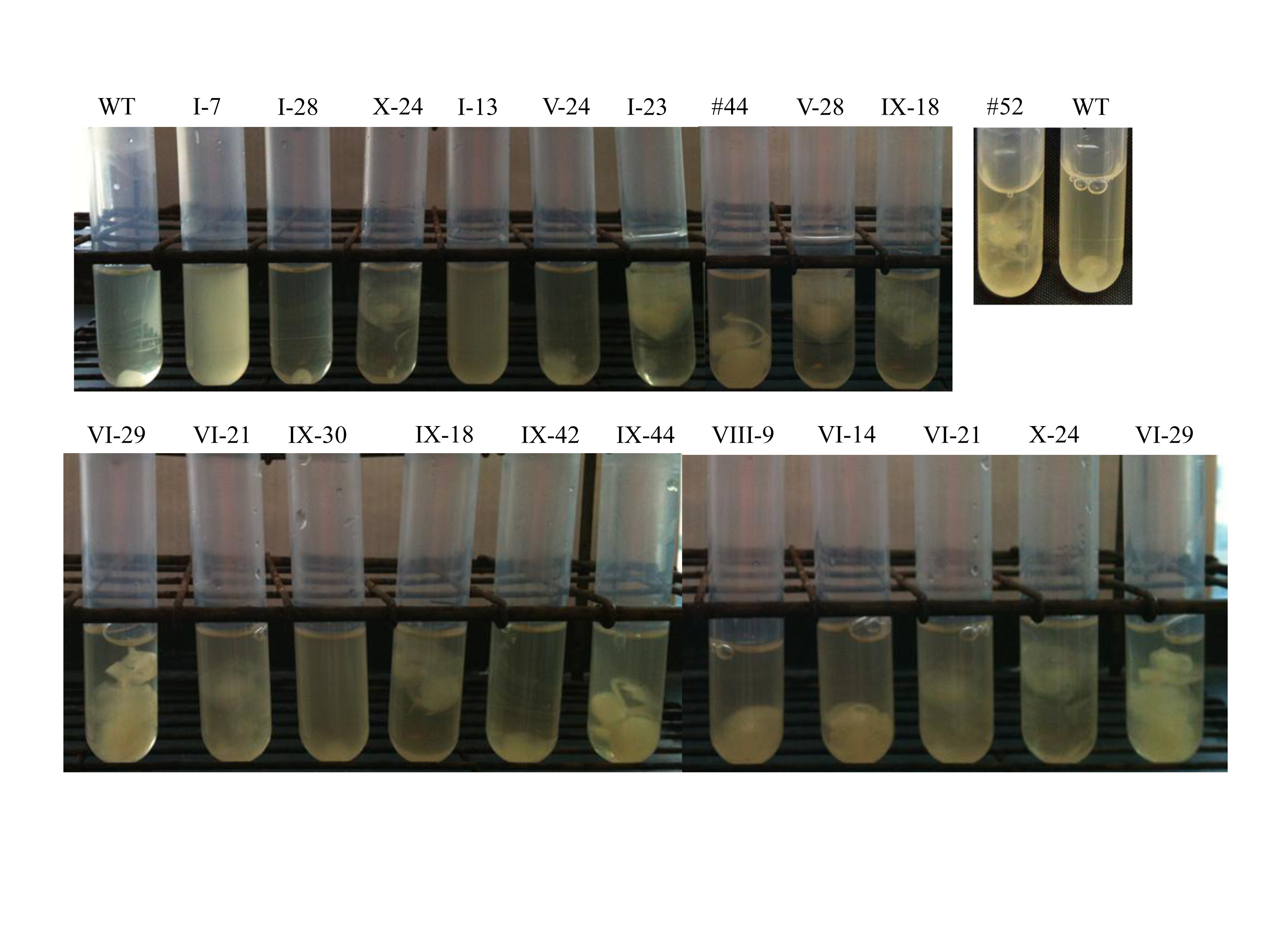

Supplement: S1 Fig — The sequences of two of the regions inserted into pUCD2 are shown below the plasmid. Genes encoding lysine decarboxylase and alanine racemase are separately ligated into the BglII and SwaI restriction enzyme sites for expression in mutants I-23 and #52, respectively. Tacp: Tac promoter; RBS: ribosome binding site; Terminator, transcription terminator. (TIF) [file pone.0119504.s001.tif]

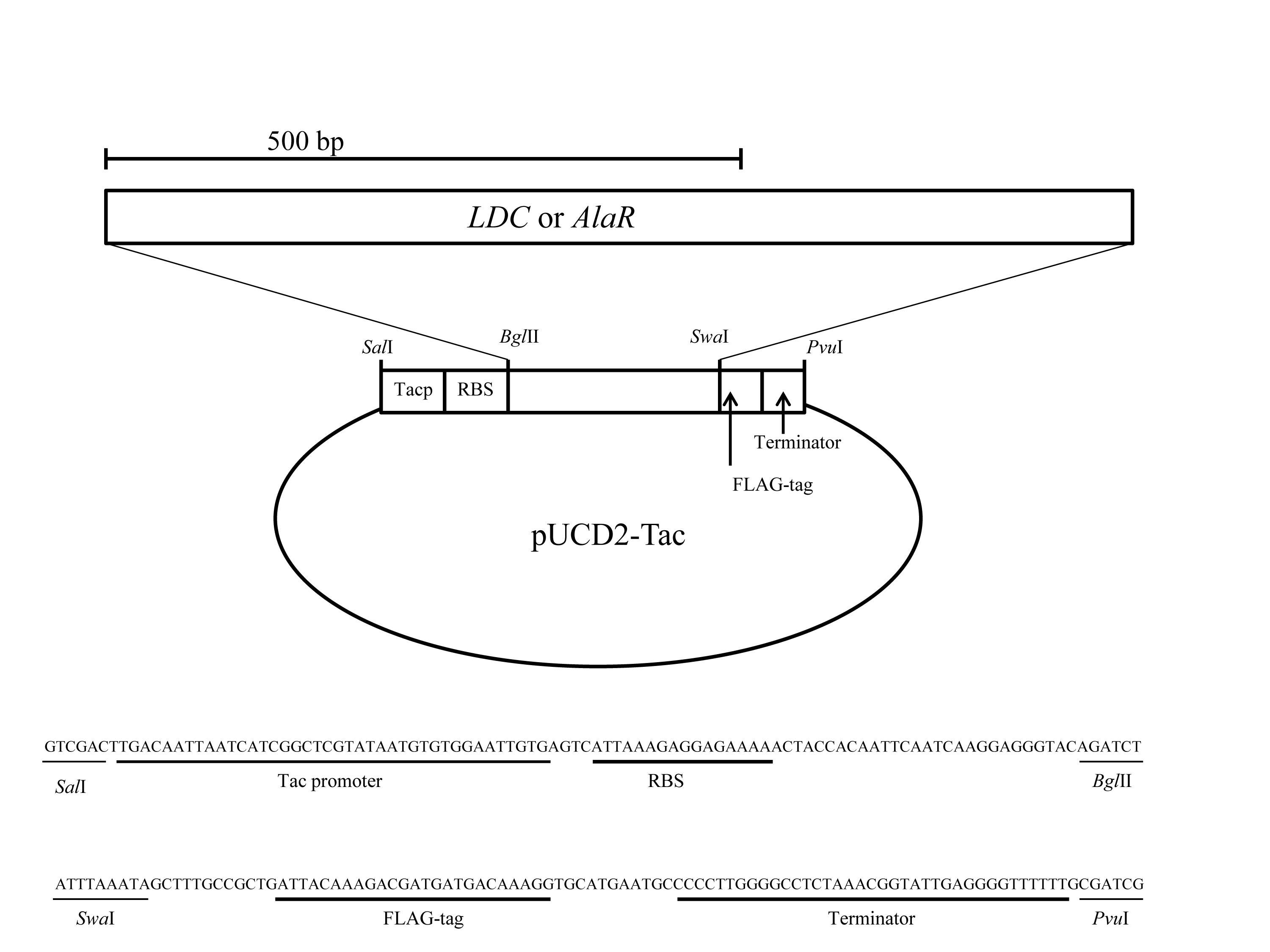

Supplement: S2 Fig — Cellulose produced was examined after growth in medium for 2 days under shaking conditions. WT: wild type; I-7 and I-13: non-cellulose-producing mutants used as negative controls [17]. (TIF) [file pone.0119504.s002.tif]

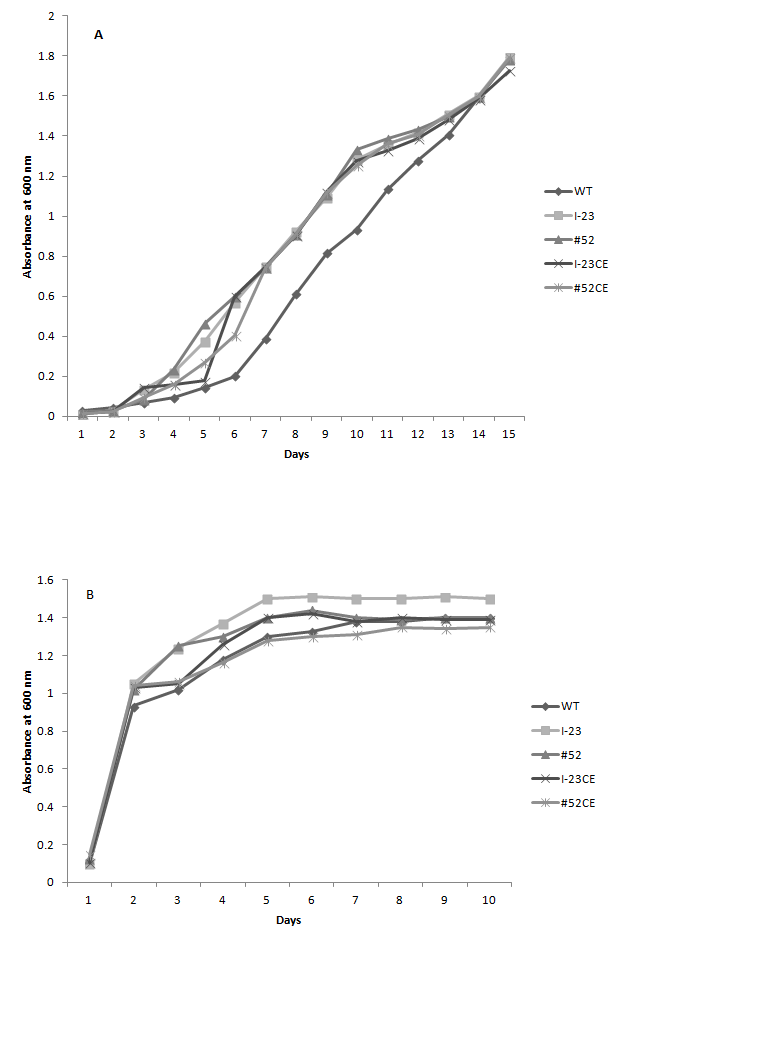

Supplement: S3 Fig — Cells were grown in 100 ml SH medium with cellulase (0.02%) under static conditions for 15 days and under shaking conditions for 10 days; for I-23 and #52, tetracycline (20 μg/ml) was also added to the medium; and for I-23CE and #52CE, both tetracycline (20 μg/ml) and spectinomycin (100 μg/ml) were added to the medium. At the same time on each day, OD600 values were taken for each culture. (TIF) [file pone.0119504.s003.tif]

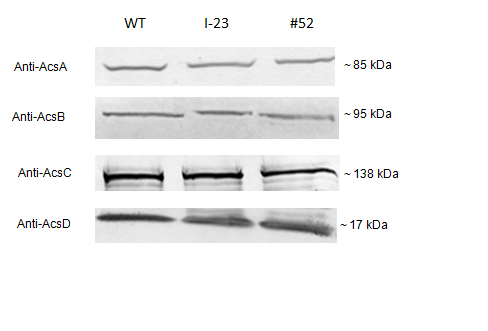

Supplement: S4 Fig — Total protein (40 μg) was loaded in each lane of 12% SDS-polyacrylamide gels. The antibody used for each blot is shown to the left, and the expected molecular mass of each protein is shown to the right. (TIF) [file pone.0119504.s004.tif]
