# Supplementary material for: Isolation and Characterization of Two Cellulose Morphology Mutants of Gluconacetobacter hansenii ATCC23769 Producing Cellulose with Lower Crystallinity
Source: PLoS One. 2015 Mar 19;10(3):e0119504. doi: 10.1371/journal.pone.0119504 (PMC4366249; doi:10.1371/journal.pone.0119504)
Supplement: S1 Table — (PDF) [file pone.0119504.s005.pdf]

Table S1 Bacterial strains and plasmids used in this study.

Strain or plasmid	Relevant genotype or characteristics	Source
Strain		
Gluconacetobacter hansenii ATCC23769	Wild type, cellulose-overproducing isolate	Deng et al. 2013 [17]
Echerichia coli Stellar^{TM}	Used for standard DNA manipulations; F-, endA1, supE44, thi-1,recA1, relA1, gyrA96, phoA, Φ80d lacZΔ M15, Δ(laZYA –arF) U169, Δ(mrr –hsdRMS –mcrBC), mcrA, λ-	Clontech
Plasmid		
pMOD^{TM}-3	EZ-Tn5^{TM} transposon construction vector; ampR, R6Kγ ori/MCS	Epicentre Biotechnology
pUCD2	Shuttle vector for *E. coli* and *Gluconacetobacter*; ampR, kanR, tetR, spcR	ATCC 37342
pGEM®-T	Cloning vector for PCR products; ampR, *lacZ*/MCS	Promega
pUCD2-Tac	pUCD2 modified to contain Tac promoter, ribosome binding site, flag-tag and transcription terminator; kanR, spcR	This work
