# Supplementary material for: Isolation and Characterization of Two Cellulose Morphology Mutants of Gluconacetobacter hansenii ATCC23769 Producing Cellulose with Lower Crystallinity
Source: PLoS One. 2015 Mar 19;10(3):e0119504. doi: 10.1371/journal.pone.0119504 (PMC4366249; doi:10.1371/journal.pone.0119504)
Supplement: S2 Table — (PDF) [file pone.0119504.s006.pdf]

Table S2 Primers used in this study.

Primer	Sequence (5′-3′)[a]	Note
TetR F1	agtcaagcttAGCTTTAATGCGGTAGT (*Hind*III)	amplification of *tetC* for cloning into pMOD-3
TetR R1	agtcgaattcGGTTGGTTTGCGCATTCACA (*EcoR*I)	
ME plus 9-3' primer	CTGTCTCTTATACACATCTCAACCATCA	For amplification of Tn5 transposon DNA
ME plus 9-5' primer	CTGTCTCTTATACACATCTCAACCCTGA	
TSP1	GAACGGGTTGGCATGGATTGT	for genomic DNA walking PCR
TSP2	GGCCACCTCGACCTGAATGGAA	
I23-CEF	aggagggtacagatctATGTCCATTTCCTCGGTCGCTGT (*Bgl*II)	for complementation of *LDC* in mutant I-23
I23-CER	gcggcaaagctatttaaatcGAAATGACCGACATTGGCGTTG (*Swa*I)	
#52-CEF	aggagggtacagatctATGTCCATTTCCATTGATATGCTCGAT (*Bgl*II)	for complementation of *AlaR* in mutant #52
#52-CER	gcggcaaagctatttaaatcGCTTACACCACCACGTGCGATGA (*Swa*I)	

[a] Lower-case letters of a primer sequence indicate the sequence designed for overlap between the resulting PCR fragment and another PCR fragment, or a cloning vector, for in-fusion cloning; upper-case letters of a primer sequence indicate the sequence corresponding to the gene to be amplified; the underlined in a primer sequence indicates the recognition sequence of the restriction enzyme shown in parentheses.
